# Training healthcare professionals in assessment of health needs in older adults living at home: a scoping review

**DOI:** 10.1186/s12909-024-06014-9

**Published:** 2024-09-17

**Authors:** Bente Hamre Larsen, Dagrunn Nåden Dyrstad, Helle K. Falkenberg, Peter Dieckmann, Marianne Storm

**Affiliations:** 1 Faculty of Health Sciences, Department of Public Health, University of Stavanger, Postbox 8600, Stavanger, 4036 Norway; 2 Faculty of Health Sciences, Department of Quality and Health Technology, University of Stavanger, Stavanger, Norway; 3 National Centre for Optics, Vision and Eye Care, Faculty of Health and Social Sciences, University of South-Eastern Norway, Kongsberg, Norway; 4 USN Research Group of Older Peoples’ Health, University of South-Eastern Norway, Drammen, Norway; 5 Center for Human Resources and Education, Copenhagen Academy for Medical Education and Simulation (CAMES), Capital Region of Denmark, Copenhagen, Denmark; 6 Department of Public Health, Copenhagen University, Copenhagen, Denmark; 7 Faculty of Health Sciences and Social Care, Molde University College, Molde, Norway; 8 Research Department, Research Group of Nursing and Health Sciences, Stavanger University Hospital, Stavanger, Norway

**Keywords:** Education, Training, Healthcare professionals, Assessment, Health needs, Home-living older adults, Healthy aging, Scoping review

## Abstract

**Background:**

Interprofessional assessment and management of health needs for older adults living at home can help prioritize community service resources and enhance health, yet there is a shortage of professionals with the necessary competencies. Therefore, support and training for healthcare professionals in community settings to assess older adults’ health with the aim of for health promotion are needed.

**Aim:**

To identify and provide an overview of published papers describing approaches for training healthcare professionals in assessing physical, mental, and social health needs in older adults living at home.

**Method:**

A systematic literature search of the Cinahl, Medline, Academic Search Ultimate, Scopus, Embase, and British Nursing Index databases was performed. We considered studies focusing on the training of healthcare professionals in assessing a single or multiple health needs of older adults aged 65 and above living at home. We considered studies published between 1990 – and March 2024. The review evaluated qualitative, quantitative, and mixed methods studies published in English-language peer-reviewed academic journals. A quality appraisal was conducted via the Mixed Methods Appraisal Tool (MMAT).

**Results:**

Twenty-three studies focused on training healthcare professionals to assess health needs and plan care for older adults living at home were included. The majority of the included studies combined teacher-driven pedagogical approaches consisting of educational sessions, written materials or e-learning, and more participant-engaging pedagogical approaches such as knowledge exchange or various forms of interactive learning. Healthcare professionals were trained to detect and manage single and multiple health needs, and some studies additionally incorporated interprofessional collaboration. Healthcare professionals were satisfied with the training content and it increased their confidence and competencies in health needs assessment and care planning for older adults. Moreover, some studies have reported that training interventions foster the implementation of new and effective ways of working and lead to positive outcomes for older adults.

**Conclusion:**

Healthcare professionals were satisfied with a combination of participant-engaging and teacher-driven pedagogical approaches used to train them in assessing health needs and planning care for older adults living at home. Such training can lead to enhanced assessment skills and facilitate improvements in practice and health promotion for older adults. Future research is recommended on interprofessional simulation training for conducting structured and comprehensive health needs assessments of older adults living at home, as well as on the implementation of such assessments and health-promoting interventions.

**Supplementary Information:**

The online version contains supplementary material available at 10.1186/s12909-024-06014-9.

## Introduction

The globally growing and diverse aging population will impact the sustainability of healthcare systems and the independent living of older adults. To support the health needs of older adults, the World Health Organization (WHO) underscores the necessity of effectively training the healthcare workforce [1, 2]. However, the complexity of health needs in older adults, coupled with an increased risk of frailty and adverse health outcomes, challenges the provision of tailored care [3]. Healthcare professionals in homecare settings are well-positioned to assess the health needs of home-living older adults [4, 5] and facilitate the interprofessional management of these needs within the community [5].

Health needs assessment should offer a comprehensive understanding of individuals’ physical, mental, and social health needs, and address the constantly changing needs with increasing age. The assessments aim to identify those who can benefit from healthcare services, such as health education, disease prevention, treatment, and rehabilitation [6]. The assessment can help set service priorities and allocate service resources effectively, guide clinical decision-making [7] and design targeted, health promoting interventions [4, 7–9] to prevent or delay frailty [10], enhance overall outcomes for those with complex health needs [11] and enable them to remain at home for as long as possible [12]. Given its importance, the task of health needs assessment, is becoming increasingly crucial in homecare settings [13]. However, there is a scarcity of adequately trained professionals proficient in conducting interprofessional health needs assessments [4, 5, 9, 14, 15], including depression [16], cognitive function [17], social needs [18], sensory function (i.e. hearing and vision) [19], geriatric healthcare [20, 21], and multidimensional frailty [22, 23]. Frailty, as a dynamic state, affects an individual who experiences losses in one or more domains of human functioning (physical, mental, social) that are caused by the influence of a range of variables, and which increase the risk of adverse outcomes [24].

A comprehensive understanding of how to train healthcare professionals in health needs assessment of the physical, mental, and social health needs of older adults living at home is crucial. This review understands training as “planned and systematic activities designed to promote the acquisition of the knowledge, skills, and attitudes” [25, p77]. Training can take place as “on-the-job training,” with practicing tasks with a mentor or receiving feedback, or through “off-the-job training,” in a classroom setting with lectures, discussions, and exercises [26]. It is essential to consistently update and expand knowledge and skills throughout healthcare professionals’ careers [27]. Mentorship and support are highly valued as pedagogical approaches [28]. Another approach is implementing interprofessional team-based training [29] focused on health needs assessment for older adults, which can be complemented by practical, supervised training with a mentor in real-world settings [9]. Interprofessional simulation training can support healthcare professionals developing communication and collaborative skills and improving patient outcomes [2]. Additionally, opportunities to share and exchange experiences and new learning with peers and seniors, along with tailored, role-focused teaching, are effective approaches training strategies in community healthcare [30]. Practical training through simulation, case studies, and role-playing influences skill development by creating experiences that promote individual understanding and learning [31] and it is based on Vygotsky’s sociocultural learning theory [32]. Tailored simulation training in use of systematic assessment tools enhanced nurses’ competencies to assess and treat complex symptoms among older adults in long-term care facilities [33].

Therefore, this scoping review aimed to identify and provide an overview of published papers describing approaches for training healthcare professionals in assessing physical, mental, and social health needs in older adults living at home. Three research questions guided the review: (1) what pedagogical approaches are used when training healthcare professionals to assess the health needs of older adults living at home, (2) what is the content and foci in the health needs assessment training provided in the studies, and (3) what are the outcomes of training reported by healthcare professionals and older adults living at home?

## Methods

### Scoping review design

This study followed the Joanna Briggs Institute (JBI) methodology [34] for conducting and reporting scoping reviews built on Arksey and O’Malley’s framework [35]: (1) Define and align the objectives (2) develop and align the inclusion criteria with the objectives (3) describe the planned approach to evidence searching, selection, data extraction, and presentation of the evidence (4) search for the evidence (5) select the evidence (6) extract the evidence (7) analyze the evidence (8) present the results (9) summarize the evidence in relation to the purpose of the review, draw conclusions and note the implications of the findings [36]. In addition, the PRISMA-ScR [37] was used as a checklist to report the scoping review data charting, data synthesis and presentation of the data (Additional file 1).

### Selection of studies

To be eligible for inclusion in the review, the study had to focus on the training of healthcare professionals in assessing physical, mental and social health needs [24], specifically assessing frailty, physical function, depression, cognition, social health, and sensory function of older adults aged 65 and above living at home [38]. Healthcare professionals from diverse fields were included, whether engaging in one-to-one interactions where individual healthcare professionals work directly with patients or working collaboratively in interprofessional teams of members from different professional backgrounds [29]. The review included qualitative, quantitative, and mixed methods studies published in English-language peer-reviewed academic journals. The inclusion and exclusion criteria are specified in Table 1 below.

**Table 1:** Eligibility criteria

Inclusion criteria	Exclusion criteria
Healthcare professionals and interprofessional teams (i.e. nurses, occupational therapists, physical therapists, social workers, care workers/assistants)	Students (bachelor and medical degree) General practitioners
Older adults (age over 65) Home dwelling/Municipal setting	Children and adults age under 65 Hospital setting or residential care facilities, palliative care.
Training in assessment of health needs, i.e. physical, mental and social, specifically assessing frailty, physical function, depression, cognition, social health, and sensory function	Training in assessment of a specific medical conditions
Peer-reviewed, empirical qualitative, quantitative, and mixed methods studies	Books, book-chapters, gray literature, literature reviews, study protocols, conference and poster abstracts and papers, media review, editorials, discussion papers, practice reports and commentary.
English languages	Other languages than English

### Search strategy

The authors and an experienced research librarian collaboratively developed the search strategy and search terms. The search strategy followed the recommendation of JBI [34]. In June 2022, a limited search of PubMed and CINAHL was conducted to identify relevant articles. To develop a more comprehensive search strategy, we subsequently analyzed the titles and abstracts of the retrieved papers, as well as the index terms used to describe the articles. A systematic literature search was performed on October 6, 2022, in the CINAHL (EBSCO), MEDLINE (EBSCO), Academic Search Ultimate (EBSCO), Scopus (Elsevier), Embase (OVID) and British Nursing Index (ProQuest) databases. The updated search was conducted on the 7th of March 2024. The search terms employed in the different databases to represent training healthcare professionals to assess health needs in older adults living at home are described in Table 2. We considered studies published between 1990 – and March 2024. Ultimately, the reference lists of all included studies were reviewed to identify any additional studies aligned with the scoping review’s aim.

**Table 2:** Search terms employed in the different databases

(assess* OR checklist* OR “check list*” OR detect* OR test* OR measur* OR screen* OR identif* OR recogniz* OR scor* OR mapping*)
AND (functional N1 (disabilit* OR declin* OR performance* OR status OR capacit* OR impair* OR limit* OR problem* OR “at-risk” OR “risk factor*” OR “cognitive function*” OR cognition OR frailty OR fragility OR sensory OR vision OR hearing OR sight OR “psychological health” OR (mental N2 health) OR “physical status” OR “physical wellbeing” OR “physical well-being” OR “loneliness” OR depressi*))
AND (education OR training OR develop* OR competence* OR learn* OR teach* OR “capacity building” OR coaching OR supervis* OR simulati*)
AND (elder* OR eldest OR frail* OR geriatri* OR “old age*” OR “old people” OR “oldest old*” OR senior* OR senium OR “very old*” OR septuagenarian* OR octagenarian* OR octogenarian* OR nonagenarian* OR centarian* OR centenarian* OR supercentenarian* OR “older people” OR “older subject*” OR “older patient*” OR “older age*” OR “older adult*” OR “older man” OR “older men” OR “older male” OR “older woman” OR “older women” OR “older female” OR “older population*” OR “older person*”)
AND (nurs* OR careworker* OR “care worker*” OR “physical therapist*” OR “occupational therapist*” OR “social worker* OR health* N1 (professional OR personnel OR worker* OR provider* or assistant*))
AND (communit* or home* or residen* or (independent* N1 living)

### Identification and selection of studies

**Fig. 1:**
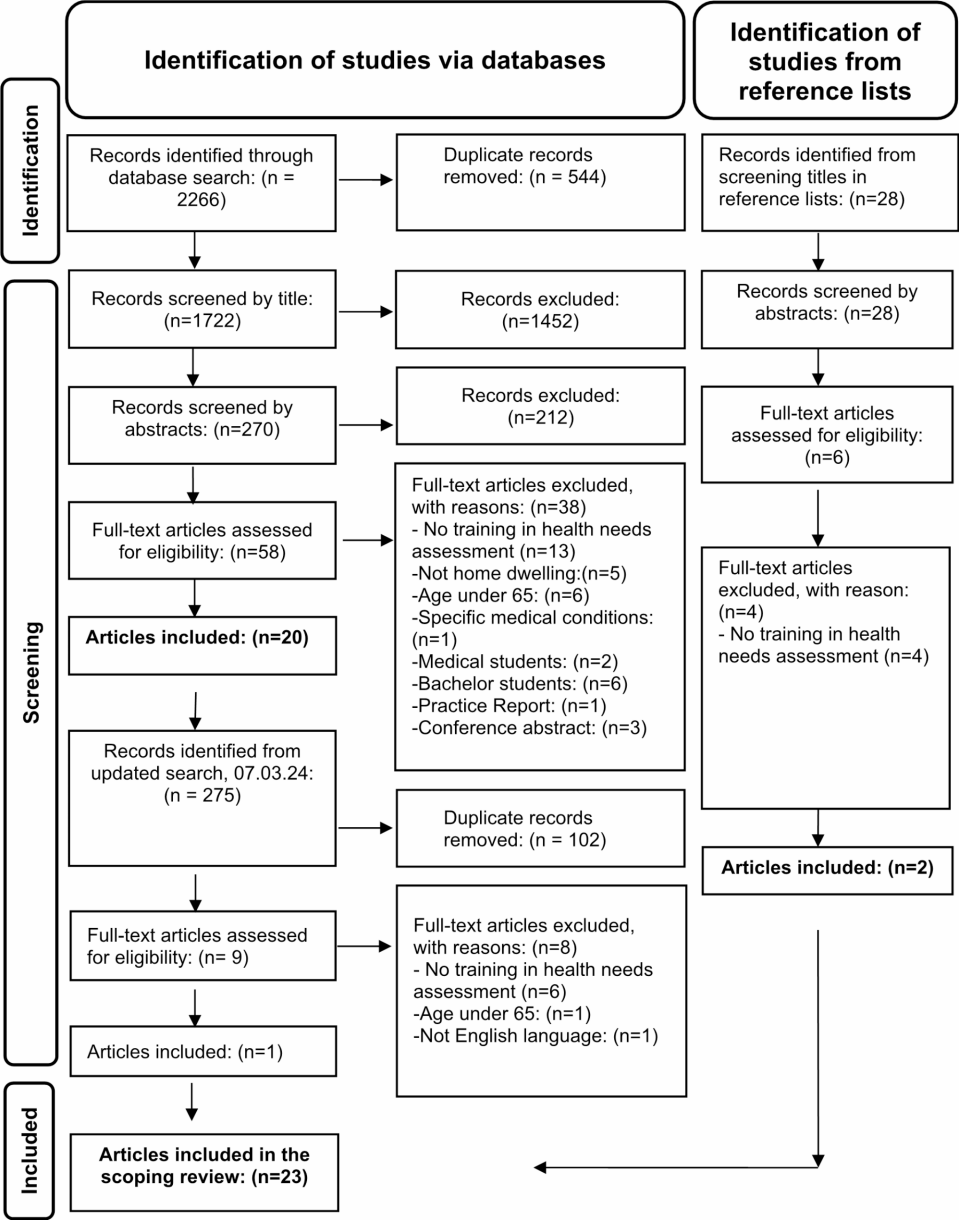
Search results, study selection and inclusion process [39]

The search yielded a total of 2266 records. The study selection process is illustrated in Fig. 1 according to the PRISMA (Preferred Reporting Items for Systematic Reviews and Meta-Analyses) flow diagram [39]. The search results were uploaded into the citation management system EndNote, where duplicates were removed. A total of 1722 records remained for screening. We used the web application Rayyan [40] to screen studies for inclusion or exclusion. The screening involved all the authors working in pairs, independently assessing eligibility on the basis of the inclusion and exclusion criteria. Discrepancies were resolved through discussions until consensus by all authors in arranged meetings.

All the records were independently screened by the authors (BHL, DND, HKF, PD and MS), and 1452 records were excluded. Two hundred seventy abstracts were reviewed in blinded pairs, leading to the exclusion of 212 records. Next, the full texts of 58 studies were read. This process resulted in the exclusion of 38 studies whose reasons are provided in the flow chart. The remaining 20 studies were included in this review (Fig. 1).

The primary reason for exclusion was the lack of content related to training in health needs assessment  (*n* = 13) or incorrect populations (*n* = 12). Eight studies were excluded because they focused on training for medical or bachelor’s degree students. Additionally, four publications were not peer-reviewed studies (*n* = 4).

BHL and MS independently screened the reference lists from the 20 included studies to identify additional eligible studies. After all the blinded titles were read, 28 titles of records were identified for abstract review. Following this, 22 titles were excluded, leaving 6 abstracts included in the full-text examination. The full-text reading further excluded four studies because they did not focus on training in health needs assessment. Finally, two studies [41, 42] were added to this scoping review, resulting in a total of 22 included studies.

An updated search was conducted on the 7th of March 2024, including publications from 2022 to 2024, following the procedure above. After removing duplicates, 173 titles and abstracts were screened for eligibility. The full texts of nine articles were read. Six studies were excluded because they did not include training for healthcare professionals. One had incorrect population, and the others were in a language other than English. This led to the inclusion of one new study [43], bringing the total number of included studies for the scoping review to 23.

### Extraction and analysis of the data

In line with the updated JBI methodological guidelines for scoping reviews [34], we extracted and coded descriptive details from the 23 included records. The extraction table covered the publication year, country of origin, study purpose, research design, study population, context/setting, training intervention content and assessment tools, pedagogical approaches and training duration, and outcomes for healthcare professionals and older adults. A test was conducted to ensure that the coauthors were aligned in their understanding of what type of data to extract for the table. Feedback from the test guided essential refinements to the extraction table before the authors collaborated to extract and organize pertinent information. We applied a basic thematic analysis to code the data and identify, analyze, and interpret patterns, ultimately deriving themes that addressed our research questions [44, 45]. The analysis utilized NVivo 12 Pro software [46].

### Quality appraisal

We performed a quality evaluation of the included studies via the Mixed Methods Appraisal Tool (MMAT) in blinded pairs. This tool is designed for a structured and standardized evaluation of methodological quality and risk of bias in systematic reviews that include qualitative, quantitative, and mixed methods studies [47]. Although quality evaluation is optional in a scoping review, it can provide valuable insights [48] and enhance the interpretability of the included studies [49].

All studies were evaluated according to five quality criteria specific to each research design (qualitative, quantitative descriptive, nonrandomized, randomized, and mixed methods studies). Each criterion received a response score of either “Yes,” indicating that the study met the quality criteria, or “No,” indicating that it did not meet the quality criteria or that it was unclear (see Table 4). It is discouraged to calculate an overall score. Any disagreements in scoring were resolved through discussion. The quality scores were not used to exclude articles from the review; instead, they were reported and discussed [49].

## Results

In accordance with the JBI scoping review guidance [44], the extracted data are presented in a table format (Tables 3 and 5) and a narrative summary is provided to respond to the three research questions. Table 3 provides a description of the study characteristics, while Table 5 outlines overarching categories along with relevant extracted information [44].

**Table 3:** Overview of the study design and study characteristics

Authors; published year; Country of Origin	Study purpose	Research design and methods	Study sample	Setting
Abassi et al. [55], 2021, Canada.	To describe and evaluate a senior community hub model aiming to better meet the health and social needs of older adults and caregivers.	Quantitative, quasi experimental one group Pre- and posttest design	**Professionals**: Multidisciplinary team, e.g. nurses, pharmacists, dietitians, kinesiologists, social workers. **Older adults**: 88 older adults < 65 years, at risk for frailty	Seniors’ community hub (model) in an academic clinic, primary care setting.
Brown et al. [52], 2010, United States	To describe the rationale, development, and use of TRIAD (depression detection) as it was implemented in a randomized controlled trail.	Pre- and posttest survey, with control group.	**Professionals**: 36 homecare nurses. **Older adults**: Medically ill, older homecare patients.	Three home healthcare agencies in Westchester County, New York
Bruce et al. [48], 2007, United States	To determine whether an educational intervention enhances the accuracy of depression assessment and the appropriateness of subsequent referrals, and to test if these referrals lead to improvements in depression.	Randomized Controlled Trial (RCT)	**Professionals**: 53 medical/surgical nurses. **Older adults**: 233 of 256 enrolled patients aged > 65 completed follow-up interviews.	Three certifies home healthcare agencies in Westchester County, New York
Brymer et al. [54], 1998, Canada	To address the belief that the geriatric assessment skills of nonphysician healthcare professionals in a rural Canadian county can be improved through a countywide educational intervention program tailored to their specific educational needs.	Pre- and posttest design.	**Professionals**: 165 health care professionals (hospital nurses, rehabilitation staff, occupational therapists, physiotherapists, social workers, homecare nurses and case managers) **Older adults**: N/A	Three hospitals in a large rural county in Ontario. All healthcare professionals were welcomed to attend, but the sessions were primarily geared to hospital nurses.
Butler and Quayle [61], 2007, Ireland	To assess the impact of the training intervention on nurses’ attitudes and knowledge toward depression in the elderly and assess the uptake of a screening protocol in practice following training.	Uncontrolled Pre- and posttest design.	**Professionals**: 253 primary care nurses: 98 nurses (74 attended one day program, 67 completed two days training). **Older adults**: N/A	Primary care, within one health services executive region in Ireland.
Couser et al. [49], 1990, United States	Description of an innovative model: geriatric assessment skills building program for community nurse practitioners.	Questionnaire evaluation	**Professionals**: 40 CH nurses **Older adults**: N/A	A course at a university for CHW nurses in the community setting working with frail elderly.
Delany et al. [23], 2011, United States	To report on and evaluate the training program for Connecticut homecare professionals, and to assess participants’ knowledge and attitudes toward depression screening and intervention in older homecare patients.	Pre- and posttest design.	**Professionals**: 25 homecare professionals. Predominantly nurses (68%). **Older adults**: N/A	14 agencies delivering homecare services in Connecticut
Landi et al. [62], 1996, Italy	To conduct a four-week training course for future care managers on the use of the National Resident Assessment Instrument (RAI), and the long-term care program application and assessment form (RAP).	Description of a training course and Statistical analysis.	**Professionals**: 14 field nurses/case mangers **Older adults**: N/A	Social services and Health services, Nursing home and Local care agency in Italy
Luptak et al. [50], 2008, United States	To educate internal staff about assessing and treating depression in older adults, and to develop an implementation process for geriatric depression care.	Survey evaluation	**Professionals**: 56 staff (multidisciplinary) from 45 sites **Older adults**: 135	Rural primary care clinics
Marcus et al. [51], 2006, United States	To train professional homecare staff to better identify persons with depression, and integrate psychiatric services within homecare agency.	Case-presentation and discussion	**Professionals**: Multidisciplinary homecare staff (nurses, physical therapists, speech therapists and occupational therapists) **Older adults**: 5024 patients, age ranged from 18–98 years.	Large urban hospital-affiliated agency and homecare agency.
Mayall et al. [39], 2004, United Kingdom	To develop and pilot a training course aimed at improving the ability of health professionals and those in the voluntary sector to detect and manage depression in older adults.	Pre- and posttest design.	**Professionals**: 40 professionals: nurses (*n* = 14), social workers (*n* = 13), GPs (*n* = 5), age concerns (*n* = 4), assistant psychologists (*n* = 2), medical students (*n* = 2). **Older adults**: Older persons.	Primary care
McCabe et al. [56], 2008, Australia	To evaluate the effectiveness of a training program to assist carers to better recognize depression among older people in both community and residential care settings	Pre- and posttest questionnaire. No control groups.	**Professionals**: 52 professional carers: registered nurse (*n* = 10), personal care assistance (*n* = 16), care managers (*n* = 13), direct carers (*n* = 13). **Older adults**: Care recipient.	Aged care in community and residential setting
Mellor et al. [57], 2010, Australia	To determine if a beyond blue depression training program for aged care workers leads to increases in knowledge, self-efficacy, attitudes, and changes in referral for depression.	Randomized Controlled trail (RCT). Pre- and posttest design.	**Professionals**: 244 professional carers from: Community care (*n* = 53) Residential (*n* = 191). Intervention group (*n* = 148), control group (*n* = 96). **Older adults**: Care recipient.	Aged care community and residential setting
Naughton et al. [59], 2016, United Kingdom	To describe the philosophical underpinnings and development of a specialized fellowship program in older person’s nursing, and report on an early-stage evaluation.	Mixed method: Qualitative focus group interview and survey.	**Professionals**: 24 senior nurses, from mental health (*n* = 4), primary/community care (*n* = 9) and acute care (*n* = 11) **Older adults**: N/A	Older Person’s Nurse Fellowship, Master program. 2014/2015, Health education in England
Neto et al. [63], 2021, Brazil	To assess the feasibility of a training intervention to enhance Community Health Workers’ capacity in addressing health and social care needs of dependent older people in deprived urban neighborhoods.	Process evaluation. Pre- and posttest.	**Professionals**: 76 Community Health Workers (CHW); 72 participated in classroom-based sessions, 58 participated in the practical home visits. **Older adults**: N/A	Homecare for dependent older persons in Brazilian city of Fortaleza.
Nunn et al. [58], 2007, Australia	To examine the feasibility and assess the experience of district nurses in screening older clients for depression.	Action Research Two cycles feasibility study.	**Professionals**: 46 district nurses. **Older adults**: aged 65 or older	District Nursing Centre
Piau et al. [64], 2013, France	To examine the feasibility and evaluate district nurses’ experience in screening older clients for depression.	Quantitative. (Letter to the editor).	**Professionals**: 63 nurses **Older adults**: 85 older adults > 75 years.	In-home assessment and care of frail older adults. Training program provided by a university hospital.
Quijano et al. [40], 2007 United States	To evaluate the Healthy Ideas Program, an evidence-based intervention for depression delivered by case managers to high-risk, diverse older adults.	Pre- and posttest	**Professionals**: 3 agency supervisors and 15 case managers **Older adults**: 172 new clients > 60 years.	Community-based service agencies and social service agencies.
Quinlan and Ryer [41], 2023 United States	To pilot an expanded primary care team model aimed at conducting fall prevention assessments and planning for older adults, facilitated by ambulatory care registered nurses during Annual Wellness visits.	A quality improvement project, reporting by the framework “Revised standards for Quality Improvement Reporting Excellence (SQUIRE)	**Professionals**: 7 Nurses **Older adults**: 522 adults at > 65 years	Ambulatory care in two urban outpatient clinics, in primary care setting.
Sin et al. [65], 2018, Singapore	To measure the perceived stress and confidence of community eldercare workers in caring for older adults with mental illness before, immediately after, and three months following their participation in a standardized training workshop on dementia and depression.	Naturalistic study. Pre- and posttest survey	**Professionals**: Multidisciplinary teams: 71 staff members **Older adults**: N/A	Various eldercare centers (daycare) and nursing home.
Smith et al. [60], 2018, United Kingdom	To evaluate the impact of a brief educational intervention on sensory impairment (hearing and sight) among community nurses.	Mixed method, Longitudinal design, Pre- and posttest questionnaire. Focus group interview post workshop.	**Professionals**: 41 community-based healthcare professionals (mainly nurses) **Older adults**: N/A	Remote island, community health setting in the western isles of Scotland.
Stolee et al. [53], 2002, Canada	To describe the implementation and evaluation of a project to develop an enhanced role in comprehensive geriatric assessment for nurses working as community-based case managers.	Quantitative, nonrandomized control group study. Pre- and posttest, survey and questionnaire.	**Professionals**: Nurse case managers **Older adults**: Frail, older adults.	Interdisciplinary Outreach consultation and inpatient services in Ontario.
Van Daele et al. [66], 2014, Belgium	To explore home nurses’ attitudes and confidence in professional competence regarding depression and evaluate a minimal intervention’s impact on their ability to detect depression in patients and family caregivers.	Quantitative Quasi experimental field study. Pre- and posttest questionnaire.	**Professionals**:92 Nurses of the home nursing organization. **Older adults**: Patients and their care givers receiving home nursing.	Three departments of a home nursing organization in the Antwerp region in Belgium.

**Table 4:** Results from the mixed methods appraisal tool (MMAT) quality evaluation [49]

Studies (*n* = 23)	Methodological quality criteria and rating				
**Quantitative randomized controlled trials**	**2.1.**	**2.2**	**2.3**	**2.4**	**2.5**
Bruce et al. [50]	**No**	**Yes**	**Yes**	**Yes**	**Yes**
Mellor et al. [59]	No	No	Yes	No	No
**Quantitative nonrandomized**	**3.1**	**3.2**	**3.3**	**3.4**	**3.5**
Brymer et al. [56]	**Yes**	**Yes**	**Yes**	**No**	**Yes**
Landi et al. [64]	**No**	**Yes**	**Yes**	**Yes**	**Yes**
Mayall et al. [41]	**Yes**	**Yes**	**Yes**	**No**	**Yes**
McCabe et al. [58]	**Yes**	**Yes**	**No**	**Yes**	**Yes**
Stolee et al. [55]	**Yes**	**Yes**	**No**	**Yes**	**Yes**
Van Daele et al. [68]	**Yes**	**Yes**	**No**	**Yes**	**Yes**
Butler and Quayle [63]	No	Yes	Yes	No	Yes
Delany et al. [16]	No	No	No	No	Yes
**Quantitative descriptive**	**4.1**	**4.2**	**4.3**	**4.4**	**4.5**
Abassi et al. [57]	**Yes**	**Yes**	**Yes**	**No**	**Yes**
Brown et al. [54]	**Yes**	**Yes**	**Yes**	**No**	**Yes**
Nunn et al. [60]	**Yes**	**Yes**	**Yes**	**No**	**Yes**
Piau et al. [66]	**Yes**	**No**	**Yes**	**Yes**	**Yes**
Quijano et al. [42]	**Yes**	**Yes**	**Yes**	**No**	**Yes**
Quinlan end Ryer [43]	**Yes**	**Yes**	**Yes**	**No**	**Yes**
Sin et al. [67]	**Yes**	**Yes**	**Yes**	**No**	**Yes**
Marcus et al. [53]	Yes	Yes	Yes	No	No
Couser et al. [51]	No	No	Yes	No	No
Luptak et al. [52]	No	Yes	No	No	No
**Mixed Methods**	**5.1**	**5.2**	**5.3**	**5.4**	**5.5**
Smith et al. [62]	Yes	Yes	Yes	Yes	Yes
Naughton et al. [61]	**Yes**	**No**	**Yes**	**Yes**	**Yes**
Neto et al. [65]	Yes	No	Yes	Yes	No

### Characteristics of the included studies

Table 3 shows that the 23 studies were published between 1990- and 2023. Eight studies were conducted in the United States [16, 42, 43, 50–54], three in Canada [55–57], three in Australia [58–60], three in the United Kingdom [41, 61, 62], and one each in Ireland [63], Italy [64], Brazil [65], France [66], Singapore [67], and Belgium [68].

Ten studies meticulously examined training interventions tailored for primary nurses [43, 50, 51, 54, 55, 60, 61, 63, 66, 68], one study specifically targeted the training of community health workers [65] and another presented an educational session tailored for case managers and agency supervisors [42]. The remaining studies indicated that training was provided to interprofessional teams or various distinct professions, such as nurses, physical therapists, occupational therapists, general practitioners, social workers and psychologists [16, 41, 52, 53, 56–59, 62, 64, 67]. The study participants were in home healthcare or primary/community care [16, 41, 43, 50–66, 68], community and social services [42, 64], mental health care [61], eldercare centers/daycare [67], residential settings [58, 59, 64], hospitals [53, 56], rehabilitation [56] and acute care [61].

### Quality evaluation results

The quality appraisal procedure revealed variations in the quality of the 23 included studies. The detailed quality evaluation results for each study are presented in Table 4, and an overview of the methodological quality criteria is presented in Additional file 3.

Each study was evaluated on five criteria appropriate to its study design category. Overall, only one study, which was a mixed methods study, met al.l five quality criteria in the MMAT [62]. Additionally, one mixed methods study met four criteria [61], and another met three criteria [65]. The most common criterion that mixed methods studies failed to meet was 5.2: whether the different components of the study were effectively integrated to answer the research question. Among the quantitative randomized studies, one study met four quality criteria [50], whereas the other was of low quality, meeting only one criterion [59]. None of these studies met the quality criterion for proper randomization. In the quantitative nonrandomized studies, six met four criteria [41, 55, 56, 58, 64, 68], one met three [63], and one met only one criterion [16], indicating low quality. All studies met the criterion regarding whether the intervention was administered as intended. The most common criteria they failed to meet were 3.3: whether there were complete data and 3.4: whether confounders were accounted for in the study design and analysis. Among the descriptive studies, seven met four criteria [42, 43, 54, 57, 60, 66, 67], one met three criteria [53], and two met only one criterion [51, 52], demonstrating low quality.

The majority of these studies met the criterion regarding whether the measurements were appropriate. However, the criterion most studies did not meet (only one out of ten) was whether the risk of nonresponse bias was low. Notably, no qualitative methods studies were included in our scoping review.

### Training interventions in assessment of older adults living at home

The next section presents a narrative overview of three major themes related to the three research questions. The themes concerned the training provided for healthcare professionals in assessing the physical, mental, and social health needs of older adults living at home: pedagogical approaches, content and foci of health needs assessment training for healthcare professionals and outcomes and evaluation of health needs assessment training for healthcare professionals and older adults living at home. The findings are summarized in Table 5 [44].

**Table 5:** Overview of training interventions, assessment tools, and outcomes for healthcare professionals and older adults

Training interventions content, pedagogical approaches and duration	Assessment tools	Outcomes for healthcare professionals and older adults
**Abassi et al. [55]** educated about principles of geriatrics and team-based care for older adults with frailty, use of assessment tools and care planning. Emphasis on interprofessional core competency. **Pedagogical approaches**: Workshops, educational sessions, and ongoing education and mentorship. **Duration**: Initial workshop, with continuing education for 1.5 Years	Electronic frailty index (eFI), Multi Domain Assessment (MDA) template, 4 m gait test, EQ-5D-5 L and EQ-VAS. Care plan support (CSP) template.	**Outcomes for healthcare professionals**: Over time, health professionals better understood health needs and felt supported in addressing biopsychosocial issue. Evaluation framework helped guiding meaningful measurement. **Outcomes for older adults**: Older adults received up to 6 connections to other health providers or social/community services. After 12 months, scores showed no statistical change, but EQ-5D-5 L domain distributions indicated enhanced functioning.
**Brown et al. [52]** educated about depression, stigma, dispelling myths, assessment of depression/suicide risk, patient interviewing, communication with patients, treatment and making referrals. **Pedagogical approaches**: True-false test, self-test in assessment skills, class discussion, lecture with PowerPoint, videotape of patient‒nurse interactions, roleplay with feedback, depression toolkit and practicing assessment in real world setting. Debriefing about implementation experience. Follow up E-mail. **Duration**: 4,5 h x two sessions. 2 follow up e-mails.	OASIS depression items M0590.	**Outcomes for healthcare professionals**: Nurses rated the training positively, and the intervention group had significantly increased confidence in assessing depression after one year. **Outcomes for older adults**: N/A^a^
**Bruce et al. [48]** educated about assessment of depression symptoms; follow-up questions, observing behavioral signs, understanding complicating factors, antidepressants, and writing referrals. **Pedagogical approaches**: Instruction, role play, tool kits, and a video demonstrating different assessment scenarios. **Duration**: 4,5 h divided in two sessions, 2 follow up e-mails.	OASIS depression items (M0590 and M0600), MMSE, Chronic Disease Score, ADLs and IADLs	**Outcomes for healthcare professionals**: N/A **Outcomes for older adults**: Depressed older adults in the intervention group had better referral rates and improved depression severity over 8 weeks. Minimal training was ineffective in improving assessment or referral, with no effect on assessment documentation
**Brymer et al. [54]** educated about medication use, physical and mental status assessment, pain, alcohol/medication use, caregiver stress, risk situations, problem identification, preliminary assessment, and intervention. **Pedagogical approaches**: Education session, demonstration, case-discussions. **Duration**: 4 × 1 h seminar.	Geriatric Depression Scale (GDS), MMSE, functional assessment (nonspecific tools)	**Outcomes for healthcare professionals**: Overall scores improved by 23% from pre- to posttest. Significant improvement in physical and mental status assessment, medication use, and elder abuse. All participants rated satisfaction with the educational sessions. **Outcomes for older adults**: N/A
**Butler and Quayle [61]** educated about depression and suicide risk, detection, management and treatment, and role of different nursing services. **Pedagogical approaches**: Workshop including lectures, group discussion, case scenarios, role-play, skills training using case vignettes and written documents. **Duration**: 2 × 8 h workshop.	Two item PRIME-MD screening and SALSA	**Outcomes for healthcare professionals**: Significant knowledge and attitude shift regarding late-life depression, with increased self-efficacy in assessment. Challenges remained in self-help efficacy and elderly suicide. Some nurses began using a brief screening protocol for depression detection in clinical practice. **Outcomes for older adults**: N/A
**Couser et al. [49]** educated about covered aging and common medical complaints, mental and psychosocial status assessments, physical examinations, thorough data gathering, decision-making skills, and effective communication with colleagues. **Pedagogical approaches**: Lecture, demonstrations, supervised lab skills training using checklist. Preceptorship training. Monthly meetings with presentation and discussion. Field trips. **Duration**: Total 30 h course (lecture) and 8 h skills training.	A non-specified checklist	**Outcomes for healthcare professionals**: Enhanced assessment skills, increased assessment frequency, and advancements in nursing practice. Greater confidence and comfort in utilizing referral and consultation resources. **Outcomes for older adults**: N/A
**Delany et al. [23]** utilized the “Training in the Assessment of Depression” (TRIAD) curriculum, which included education on depression, effective interviewing and assessment techniques, follow-up questioning, interventions, and pharmacological management. **Pedagogical approaches**: Web-based training with video filmed vignettes, information, PowerPoint, experiential case study assessment practice and discussion. Downloadable toolkits. **Duration**: 4 h	PHQ-2 and PHQ-9 tools in OASIS-C.	**Outcomes for healthcare professionals**: The program received high ratings, indicating significant improved attitudes and self-efficacy in caring for older adults. Increased confidence to assess depressed older adults. No significant changes in knowledge about depression in general were noted. **Outcomes for older adults**: N/A
**Landi et al. [62]** educated about community elderly care and case management skills as assessment techniques, case management, triggering systems, and developing care plans. The RAP manual served as a comprehensive gerontological textbook. **Pedagogical approaches**: Instruction manuals and course textbook, lecture, slides, discussions, presentation of simulated cases with interactive study group discussions, assessing in real world setting with tutor, presentation of the assessment and the care plan experiences. Final exam including written cases and videotapes of actual assessment-cases discussed by a simulated interdisciplinary team. **Duration**: Four weeks training course.	RAI (Comprehensive assessment instrument) for training, and The British Colombia (B.C).	**Outcomes for healthcare professionals**: Participants got equipped with strong assessment, decision-making and care planning skills. **Outcomes for older adults**: N/A
**Luptak et al. [50]** educated about different types of depression diagnosis and criteria, various depression screening tools and instruction for its use, treatment options, follow up measures and referrals. Practical training in using the adapt screening, assessment and patient follow up materials. **Pedagogical approaches**: CD-rom with written educational material, including list of assessment tools and instructions for its use. Training session with case-based role-play, discussion, and question-and-answer period. **Duration**: 4-hour session	Geriatric Depression Scale (GDS 15) and a modified IMPACT screening instruments.	**Outcomes for healthcare professionals**: Improved knowledge and skills in depression screening, and the number of patients screened increased. Some reported earlier identification of geriatric depression, enhanced communication with primary providers, and improved follow-up. **Outcomes for older adults**: N/A
**Marcus et al. [51]** educated about how to identify and refer persons with depression and suicidal intent. **Pedagogical approaches**: Instructions and interactive learning through scripted videos featuring diverse actors and facilitated group interaction. **Duration**: N/R	PHQ-9 assessment instrument (OASIS).	**Outcomes for healthcare professionals**: Integrating psychiatric services into the home health agency improved homecare nurses’ ability to identify and address depression. Reporting screening results to the patient’s physician or mental health specialist became standard protocol for the agency. **Outcomes for older adults**: Referrals for mental health treatment significantly increased, with 75 patients referred for evaluation over a 6-month post training period. Twelve patients required consultation between social staff and psychiatrist, and 63 received diagnose.
**Mayall et al. [39]** educated on assessing and detecting depression in older individuals using the Geriatric Depression Scale (GDS-10), treatment, management, interventions, policy frameworks, and available local services. Covered user experience, and differentiation between dementia and depression. **Pedagogical approaches**: Lectures and demonstrations. **Duration**: Three 4-hour workshops	Geriatric Depression Scale (GDS10)	**Outcomes for healthcare professionals**: Participants significantly improved in knowledge, confidence, and skills post training, with 44% finding the session on assessing depression and local services particularly useful. Depression detections increased slightly, and use of the assessment tool (GDS) rose significantly. Skills in interventions and referrals were also enhanced, meeting training expectations. **Outcomes for older adults**: N/A
**McCabe et al. [56] e**ducated on depression in older adults (detection, monitoring, treatment), communication with older adults and healthcare professionals, and making appropriate referrals. **Pedagogical approaches**: Instruction and a manual with educational material, that used examples and case studies. Advanced session covered skills training and discussion. **Duration**: Basic sessions: 4 × 90 min. Advanced sessions: 2 × 2 h.	Geriatric Depression Scale (GDS-15) and Cornell Scale.	**Outcomes for healthcare professionals**: Higher knowledge of depression, increased self-efficacy in detecting and working with depressed older adults, and reduced barriers to providing care and treatment for depression, all maintained at 6 months. Nurses and care managers reported increased confidence in assessing psychiatric history and collaborating with families and medical professionals. **Outcomes for older adults**: N/A
**Mellor et al. [57]** educated about depression, detecting depression (also within older adults with dementia), monitoring the treatment response, writing referrals and communication with healthcare professionals. **Pedagogical approaches**: Training manuals, case studies, instruction, and information. Reflection and discussion. Advanced sessions: skills training. **Duration**: Basic sessions: 4 × 90 min. Advanced sessions: 2 × 2 h	Geriatric Depression Scale (GDS-15) and Cornell Scale.	**Outcomes for healthcare professionals**: Professionals in the intervention group showed increased knowledge, self-efficacy, and positive attitudes over time toward working with late-life depression. While their tendency to refer to specialists did not significantly change, there was a slight increase post training. Senior staff also gained confidence in conducting assessments and collaborating with health specialists after training. **Outcomes for older adults**: N/A
**Naughton et al. [59]** educated about quality-of-life issues, including comprehensive geriatric assessment (frailty, nutrition, mobility, pharmacology, mental health, cognitive function, continence, and end-of-life care). Covered collaborative management of complexities with older individuals, their families, and the multidisciplinary team, as well as decision-making and care planning. **Pedagogical approaches**: Flipped classroom model with prerecorded lectures and online exercises. Face to face study days with teaching. Facilitated network and peer to peer learning. **Duration**: 12-month program, once a month for face-to-face study days.	Comprehensive geriatric assessment (CGA)	**Outcomes for healthcare professionals**: Overall satisfaction was very high, particularly regarding the topics of CGA, pharmacology, and mental health. The majority expressed strong agreement on the first modules relevance, organization, and appropriate assessment. They valued peer-to-peer learning and had intention to change practice (median score 4 of 5). Nurses reported about increased referrals based on frailty screening. **Outcomes for older adults**: N/A
**Neto et al. [63]** educated about “Screening for Geriatric Risk Factors”, covering health and functional capacity in later life, ADL, dependence criteria for older persons and their families, risk situations, preventive strategies, communication with family health strategy teams and social services, basic social support and treatment, **Pedagogical approaches**: Classroom based sessions and supervised assessment in real world setting. **Duration**: 28 h classroom based session. 12 h skills training.	Screening tool of geriatric risk factors	**Outcomes for healthcare professionals**: Participants significantly increased their knowledge of older adults’ health issues and how to respond to their needs post training. They successfully conducted home visits and screened for risk factors afterward. The course received highly positive ratings for both classroom sessions and supervised home visits. **Outcomes for older adults**: N/A
**Nunn et al. [58]** educated about depressions and administration of the depression scale (GDS) and referring. **Pedagogical approaches**: Educational sessions, practice in depression-screening in real world setting, workshop using nominal group technique, role play and practical training in real world setting, with available support, information, and encouragement. **Duration**: Two-hour education session x 2	Geriatric depressions scale (GDS 5–15). .	**Outcomes for healthcare professionals**: After the first educational session, nurses had resistance in using GDS, and felt uncertain about handling positive screening results. Suggestions were given to improve the session. Following the second session, most found it useful, with only one rating it as not. Most felt prepared to use GDS and believed district nurses should conduct depression screenings. Feedback on GDS was positive, increasing awareness of depression. 62% felt it helped identify depression, while 50% felt ready to assist depressed clients. Concerns included GDS’s suitability for many clients. **Outcomes for older adults**: N/A
**Piau et al. [64]** educated about frailty and the use of geriatric and frailty assessment tools, and how to develop a first-line personalized care and prevention plan in collaboration with the general practitioner. **Pedagogical approaches**: N/R **Duration**: 40 h training program	Gerontopole frailty screening tool (GFST), Short physical performance battery (SPPB), ADL, Nutrition, Cognitive, social and sensor function (unspecified assessment tools)	**Outcomes for healthcare professionals**: Nurses identified frailty causes and effectively recommended interventions and referrals, efficiently using their expanded assessment and care planning roles. **Outcomes for older adults**: 285 patients underwent in-home geriatric assessments, resulting in changes across multiple domains. A high proportion of assessments effectively led to interventions such as physiotherapy prescriptions, oral supplement prescriptions, and referrals for geriatric consultations.
**Quijano et al. [40]** educated about screening and assessment, self-management, referral and linkage, and behavioral activation for depression. **Pedagogical approaches**: Self-study of educational material and videos. Lectures, skills demonstration, and role-play. Individual coaching 3 months post training, and a group booster session. **Duration**: Self-study and 3 half day group training. Coaching twice a month for 3 months.	Primary care evaluation of mental disorder (PRIME-MD), Geriatric depression scale (GDS-15) mini mental state exam, and items about quality of life, medical and mental health utilization, social and physical activity, and depression self-management.	**Outcomes for healthcare professionals**: The study results demonstrate that case managers could identify depression and carry out an evidence-based self-management intervention post training. Program continued after intervention period. **Outcomes for older adults**: The mean GDS-15 scores improved significantly from baseline to 6 months, indicating a reduction in depression severity and pain. Participants also demonstrated increased knowledge about seeking help and the importance of activity
**Quinlan and Ryer [41]** educated about epidemiology of older adults fall risk (gait, balance, strength, home hazards, footwear, vision and medications), age friendly health system principles, motivational interviewing, gait, strength, balance and home safety and fall prevention interventions. **Pedagogical approaches**: 15–30 min self-paced online learning modules, and 90 min live virtual training sessions **Duration**: Average total time commitment of 5 h or less	Three fall-related questions. Fall history review, Time Up and Go test (TUG) and a 13-question home safety checklist (CDC STEADI Check for Safety Brochure).	**Outcomes for healthcare professionals**: Pilot Registered Nurses voiced satisfaction with their expanded role, and the prevention program content. Program continued after intervention period. **Outcomes for older adults**: Out of 522 patients screened, 111 were identified as at risk for falls, with older age showing a correlation. Eighty-three patients worked with nurses to develop Fall Prevention Plans of Care (FPOC). Following a two-week follow-up call, most patients reported implementing their plans, with only one fall reported during this period.
**Sin et al. [65]** educated about diagnosis, identify signs and symptoms, assessment, and management of dementia and depression. **Pedagogical approaches**: Teaching, case discussions and group discussions ending with summary of key point. **Duration**: Workshop on dementia (4 h) and workshop about depression (4 h)	Rating instruments (nonspecified)	**Outcomes for healthcare professionals**: Significant improvement in knowledge of depression and dementia, and positive changes in care and understanding for older adults with these conditions. This included increased confidence, efficiency, satisfaction, and safety, improving detection, management, and patient communication. However, one participant faced challenges applying their newfound knowledge due to manpower and resource constraints. **Outcomes for older adults**: N/A
**Smith et al. [60]** sight and hearing impairments, their impact on daily living aspects, communication techniques, patient assessment referral pathways, and solutions to support individuals with sensory impairments. **Pedagogical approaches**: Interactive training workshop with simulation practice and information. **Duration**: 3–4-hours.	N/A	**Outcomes for healthcare professionals**: Improved knowledge, understanding, and awareness of sensory impairments were observed. Positive changes were noted in communication, assessment, information, and referral practices. The content and pedagogical approaches received positive evaluations, particularly regarding simulation activities. **Outcomes for older adults**: N/A
**Stolee et al. [53]** educated about dementia, depression, delirium, falls, medications: Assessment, collaboration and report writing. **Pedagogical approaches**: Lectures and discussions, self-directed learning periods, clinical experience, and interdisciplinary case conferences. In implementation phase: in-service educational sessions. **Duration**: Practicum education during a 3 weeks/15 days period and additional 35 h of clinical work over a 6 months’ period.	Comprehensive geriatric assessment – non specified assessment tool.	**Outcomes for healthcare professionals**: Significantly increased confidence in assessing dementia, depression, delirium, functional decline, Parkinson’s disease, stroke, and lack of competency. They consistently used standardized assessment tools and effectively managed health needs. Physicians found the information and recommendations useful, and the training sessions were well-received. **Outcomes for older adults**: 10 out of 13 physicians noted enhanced patient outcomes, such as home supports and placement issues.
**Van Daele et al. [66]** educated about depression, covering personal experiences, psychiatric disorders, symptoms, detecting symptoms, comorbidities, communication with patients and treatment options **Pedagogical approaches**: Information, explanation, possibility to ask questions, video recordings from staged situations followed by group discussion. **Duration**: 1 h intervention.	Screening questions by Whooley et al., (1997) and Arroll et al., (2005).	**Outcomes for healthcare professionals**: Minimal impact on attitudes and confidence in professional competence regarding depression, except for decreased confidence in their role in helping depressed patients. However, it significantly increased detections and referrals. Nurses who detected patients felt more competent than those who did not, and confidence in recognizing depression improved over time for both groups. **Outcomes for older adults**: A minimal intervention resulted in a significant increase in the number of detections of depression and referrals to GPs. 43 individuals with depressive symptoms were identified; 31 were referred to their GP

^a^ Not applicable

### Pedagogical approaches

The included studies employed diverse pedagogical approaches to train healthcare professionals in assessing the health needs of older adults living at home. The spectrum of pedagogical approaches observed in the studies was categorized into teacher-driven and participant-engaging pedagogical approaches. Twenty-one studies [16, 42, 43, 50–65, 67, 68] combined teacher-driven and participant-engaging pedagogical approaches, reflecting a multifaceted training strategy. Mayall et al. [41] opted for a more singular pedagogical approach, exclusively relying on lecture-based education, whereas the training method used in the Piau et al. [66] study remained unspecified. The training interventions varied in duration, from one-hour sessions [68] to an ongoing training program spanning 21 months [57]. In two studies, the specific duration of the training interventions was not specified [53, 57]. The most common duration for training was 4–8 h [16, 43, 50, 52, 54, 56, 58, 59, 62, 67].

### Teacher-driven pedagogical approaches

Almost all studies utilized teacher-driven pedagogical approaches, including educational sessions, written materials or e-learning [16, 41, 42, 50–65, 67, 68]. Educational sessions were evident in 14 studies [16, 41, 42, 50–53, 56–59, 62, 64, 65], providing healthcare professionals with information about relevant topics through lectures [16, 41, 42, 51, 62, 64, 65], slides [16, 64] and instructions [50, 52, 53], as well as demonstrations of the use of assessment tools [41, 42, 51, 56, 58, 59]. Additionally, Abbasi et al. [57] and Quijano et al. [42] offered ongoing sessions during the post training implementation period.

Written materials were provided to the participants in nine studies [16, 42, 50, 52, 54, 58, 59, 63, 64]. This included training manuals containing examples and case studies [58, 59], written documents about the training pack and the assessment forms [63], course textbooks and instruction manuals [64], educational materials including the program manual and articles [42], a CD-ROM (a data-disc for computer) containing written educational material [52] and toolkits derived from the educational material [16, 50, 54]. Brown et al. [54] reported that toolkits included key intervention components for seamless application of learned concepts [54]. Furthermore, some described follow-up emails to provide participants with information post training [50, 54].

E-learning as a preplaying online module or videoclip appeared in nine studies [16, 42, 43, 50, 53, 54, 61, 64, 68]. Naughton et al. [61] delivered prerecorded lectures [61], Landi et al. [64] used video recordings presenting real cases to test participants’ assessments- and decision-making skills, and Quinlan and Ryer [43] offered online modules on aging epidemiology, fall risk factors, and age-friendly health systems [43]. Participants watched video recordings portraying late-life depression [42, 53, 54], and patient interactions illustrating approaches to depression assessment [50, 54, 68] via standardized questions and follow-up questions [16]. Professional actors were used in three studies [16, 53, 68].

### Participant-engaging pedagogical approaches

The majority of the included studies utilized participant-engaging pedagogical approaches involving knowledge exchange or various forms of interactive learning [16, 42, 43, 50–65, 67, 68].

Sixteen studies employed various forms of knowledge exchange such as discussion, questioning and coaching, between training participants and teachers [43, 51–56, 58–61, 63–65, 67, 68]. Peer-to-peer learning and dialog facilitated the exchange of knowledge and insights [65], which enriched the overall learning experience [61]. The participants were included in discussions following lectures [55], after watching scripted videos [53], and during patient case reviews [56, 67]. Additionally, three studies included both discussions and allowed participants questions [52, 54, 68]. Discussions allowed participants to delve into case management techniques [51], explore experiences related to assessing the health of older adults [54, 60, 64] and solve problems and discuss alternative strategies regarding depression screening [54]. A few studies have provided ongoing coaching in the post training phase to support healthcare professionals in applying newly acquired skills [42, 55, 57]. This included three months of feedback and support [42], mentorship for skill integration [57], and a six-month collaborative approach between resource staff and case managers involving home visits and clinical consultations [55].

Interactive training was employed in fifteen studies through skills training, role-playing, simulations, and hands-on training in real-world settings [16, 42, 43, 51, 52, 54, 55, 57–60, 62–65]. Skill training allows nurses to practice patient interviews and assessments and receive instructor feedback [54]. The participants practiced by assessing their colleagues’ health and responding to assessment [16, 51], with faculty staff offering assistance, encouragement, and feedback throughout [51]. Landi et al. [64] provided practice exercises followed by presentations, and Quinlan and Ryer [43] provided a virtual training session in motivational interviewing technique and assessment. Roleplay as a teaching strategy was used to address practical aspects of administering depression screening [50, 52, 60], and Butler and Quayle [63] incorporated case scenarios, roleplay, and practical skills training for assessing depression in older adults [63]. Simulation training was used to immerse participants in the experience of living with sight and hearing impairments performing everyday tasks such as filling out forms or managing medications using sight impairment spectacles. Training was followed by a debriefing session [62]. Hands-on training in the assessment of older adults’ health in real-world settings was conducted in ten studies [42, 51, 54, 55, 57–60, 64, 65]. Healthcare professionals gained clinical experience through assessments of home dwelling older adults [55, 58–60, 64, 65] and through participation in a rotational preceptorship for community health nurses. This enabled them to practice newly acquired assessment skills and collaborate in a real-life setting [51]. Additionally, two other studies emphasized practical training in communication with other professionals in real-world settings [58, 59], while Brown et al. [54] encouraged participants to practice assessments in a real-world setting between educational sessions.

### Content and foci of health needs assessment training for health care professionals

All the included studies offered insights into the content and foci of health neesd assessment training interventions for healthcare professionals. The studies were divided into those aimed at training healthcare professionals to understand and assess either single or multiple physical, mental, and social health needs in older adults living at home. Additionally, some training sessions focused on interprofessional collaboration.

### Single health need assessment training

The focus of twelve studies involved enhancing the skills of healthcare professionals in assessing, planning and conducting interventions for a specific, single health need in older adults, with each addressing either the assessment of mental or physical health [16, 41, 43, 52–54, 58–60, 62, 63, 68]. Two of these studies [43, 62] focused solely on physical health factor training. Smith et al. [62] emphasized training in assessing and detecting sight and hearing impairments without specifying whether any assessment tools were used [62]. Quinlan and Ryer [43] provided fall risk assessment training, which included the use of assessment tools to evaluate the physical function of older adults and to assess their home environments. The other ten studies [16, 41, 52–54, 58–60, 63, 68] focused on training to assess depression in older adults living at home. The training encompassed understanding and detecting the condition, and all of them included the use of assessment tools. Van Daele et al. [68] included skills such as actively listening to patients and motivating them to seek expert assistance when needed. Delaney et al. [16] incorporated skills in asking follow-up questions, and Mellor et al. [59] offered training in appropriate communication with older adults to identify masked, early signs of depression.

### Multiple health needs assessment training

Eleven studies [42, 50, 51, 55–57, 61, 64–67] described training interventions for healthcare professionals aimed at assessing, planning, and conducting interventions for multiple health needs in older adults living at home. The training content ranged from learning to performing a holistic health assessment of older adults encompassing physical, mental, cognitive, and social factors [42, 51, 55–57, 61, 64, 66] to a more nuanced assessment of two or three of these factors [50, 65, 67]. All studies described the use of assessment tools or checklists. A holistic assessment and understanding of older adults’ health context and needs enables interventions to be tailored to their health and care needs, priorities, and levels of frailty [57]. Within the realm of holistic assessment, only two of these studies addressed alcohol and medication usage [55, 56], whereas two other studies focused on evaluating sensory status [57, 66]. For studies with more nuanced assessment training, three studies [42, 50, 67] primarily tailored their training to focus on depression assessment and intervention in older adults, but Quijano et al. [42] also included training in assessing general physical health status, social function, and cognitive function. Sin et al. [67] included dementia assessment and Bruce et al. [50] addressed factors that commonly complicate depression in homecare patients, such as health conditions, disability in activities of daily living, and cognitive function. The training included how to ask follow-up questions and observe nonverbal language [50]. Neto et al. [65] provided training for healthcare professionals in rural areas to screen for geriatric risk factors such as caregiver overburden, general health, social health, risk of falling, or difficulties in activities of daily living.

### Interprofessional collaboration and communication skills in health needs assessment training

Beyond the focus on training for assessing the health needs of older adults, sixteen studies [42, 50–55, 57–62, 64, 65, 68] have incorporated training elements to increase interprofessional collaboration and communication skills among healthcare professionals. Health needs assessment training for interprofessional teams was evident in eight of the included studies [51, 55, 57–59, 61, 64, 65]. Two studies [58, 59] outlined an advanced session to teach skills for interacting with other healthcare providers, including general practitioners and mental health specialists, whereas Couser et al. [51] stressed the importance of effectively communicating the assessment results to physicians and other healthcare providers. Training in writing referrals was emphasized in ten studies [42, 50, 52–54, 58–60, 62, 68]. In addition, Stolee et al. [55] trained healthcare professionals in writing reports and making recommendations to the referring case manager. Only two studies [61, 65] included collaboration with family in their training programs. Naughton et al. [61] designed training programs to support healthcare professionals in navigating the complexities of collaboration with multidisciplinary teams, older adults, and their families. They also developed a network among nurses to facilitate the exchange of expertise, experience, and innovative ideas [61]. Neto et al. [65] aimed to increase the capacity of care workers to effectively collaborate with family caregivers and social services for dependent older adults in rural areas. Stolee et al. [55] provided training for case managers to extend this knowledge to their teams and strengthen connections with specialized geriatric services. Similarly, Abbasi et al. [57] emphasized team-based care delivery training, with active and holistic discussions among patients, caregivers, and interprofessional teams. Diverse skill sets within teams can effectively meet the holistic care needs of patients. In parallel, Piau et al. [66] focused on training nurses to collaborate with general practitioners to develop comprehensive care plans. Landi et al. [64] trained case managers who collaborated in supervised teams to assess older adults and present care plans. They watched videos of simulated team discussions to enhance their understanding of the assessment process and teamwork [64].

### Evaluation and outcomes of health needs assessment training for healthcare professionals and older adults

All of the studies provided insight into the experiences or outcomes of healthcare professionals participating in the training interventions. This included their satisfaction and experiences with health needs assessment training, improved confidence and competencies in health assessment and care planning and shifts in work practices. Additionally, some studies have reported outcomes for older adults following health needs assessment training, such as appropriate referrals, tailored interventions, fall prevention, symptom reduction, and improved overall function. The evaluation of these outcomes relied to a small extent on models or frameworks, with only three studies incorporating them [43, 61, 62]. Smith et al. [62] utilized Kirkpatrick’s four-level training evaluation model to assess the relevance and impact of educational intervention. Naughton et al. [61] adopted Alvarez et al.’s (2004) framework of an integral model of training evaluation and effectiveness. Quinlan and Ryer [43] presented their findings following the Revised Standards for Quality Improvement Reporting Excellence (SQUIRE) framework.

### Healthcare professionals’ satisfaction and experiences with assessment training

Ten studies provided insights into healthcare professionals’ experiences with participating in training interventions [16, 41, 43, 54–56, 60–62, 65], where most of the participants expressed satisfaction with both the content and format of the courses. The participants in Brymer, Cormack and Spezowka [56] expressed a high level of satisfaction with the presenter’s content, pacing, and format, and in Mayall et al. [41], the training met the participants’ needs and expectations. The participants in Naughton et al. [61] particularly valued the peer-to-peer learning aspect, whereas Smith et al. [62] emphasized the effectiveness of simulations. Neto et al. [65] rated classroom sessions and supervised home visits very positively and found them useful. Furthermore, participants in four of the studies [16, 60, 61, 65] offered suggestions to enhance the number of educational sessions. They suggested allocating more time for training [16, 65], a greater focus on skills training [60, 61], additional training in managing complex and technically challenging issues [65] and incorporating more time for case studies and discussions [16].

### Improved confidence and competence in health assessment and care planning

Improvements in assessment competencies following training interventions among healthcare professionals were reported in nineteen studies [16, 41, 42, 51, 52, 54–60, 62–68]. Among these, nine studies explicitly reported increased confidence among healthcare professionals in assessing older adults’ health needs [16, 41, 54, 55, 58, 59, 63, 67, 68]. The health need sassessment and use of assessment tools or checklists led to the identification of health needs. Quinlan and Ryer [43] noted that without screening in a fall prevention program, the identification of fall risk among older adults would be missed. Piau et al. [66] noted that a high proportion of assessments effectively identified frailty and suggested interventions and referrals. One comment was that they “were previously skirting around the problem, now asked about mental health directly” [61, p. 33]. Naughton et al. [61] reported that performing a comprehensive geriatric assessment helped when raising issues with general practitioners because they were talking about their language. Nunn, Annells and Sims [60] acknowledged the use of Geriatric Depression Screening (GDS) tool raised awareness of depression. A total of 62.5% felt that the GDS helped identify depression that might otherwise be overlooked, but some questioned its universal usefulness [60]. Abbasi et al. [57] reported that having an evaluation framework helped healthcare professionals guide meaningful measures [57]. Conversely, some participants also expressed that they relied more on observation than direct questions when assessing depression [54]. According to Landi et al. [64], careful assessments is deemed essential for effective care planning, and Stolee et al. [55] emphasize the critical role of assessment training in identifying health needs and equitably distributing community service resources. Two studies reported one year of retention of knowledge and skills without the inclusion of a refresher course [54, 62].

Twenty studies documented a better understanding of appropriate interventions and referrals [16, 41, 42, 50–53, 55, 57–68]. According to Delaney et al. [16], 50% of the participants noted that a key aspect they learned was understanding the significance of the assessment results and the corresponding interventions [16]. The participants in the study by Neto et al. [65] demonstrated significantly improved capacity in responding to the health and care needs of older adults. The participant reported increased confidence in making referrals and consulting resources [51], increased knowledge about managing depression, making referrals, and accessing available local services [41] and enhanced self-efficacy in providing care for older adults [16, 58]. Nunn, Annells and Sims [60] reported that 50% of participants felt prepared to address older adults’ depression after training. Smith et al. [62] observed increased referral practices and improved ability to advise patients about sensory services, whereas Mellor et al. [59] noted a slight increase over time in specialist referrals, and senior staff reported increased confidence in interacting with health specialists.

### Shift in healthcare professionals’ work practices after assessment training

The training intervention resulted in either a change or potential for change in work practices in ten studies [16, 42, 43, 52, 53, 55, 57, 62–64]. Butler and Quayle [63] reported that prior to receiving training, nurses did not utilize any formal assessment measures to screen for depression in older adults. However, following training, some nurses continue to use screening measures for depression in their clinical practice [63]. Similarly, case managers in Stolee et al. [55] stated that the major change in their assessment practice was greater consistency in the use of assessment tools. Smith et al. [62] reported a shift in practice toward incorporating more detailed information about patients’ impairments and implementing supportive strategies, and in Marcus et al. [53], communication of depression screening results to patients, physicians, or mental health specialists became a standard protocol.

Landi et al. [64] reported that training was proven feasible and may be implemented on a broader scale, and Luptak et al. [52] outlined an implementation period of the ADAPT—Assuring Depression Assessment and Proactive Treatment protocol for depression care in rural healthcare—with the potential to achieve the outlined goals in various clinical settings [52]. Delaney et al. [16] reported that project participants were interested in implementing the program in their homecare setting and developed a train-the-trainer model. Abbasi et al. [57] provided results and experiences regarding the Seniors Community Hub (SCH) through the ADKAR (awareness, desire, knowledge, ability, reinforcement) evaluation framework to assist others interested in implementing a similar integrated care model [57]. Quinlan and Ryer [43] stated that fall assessment practices are currently implemented and continuous; similarly, Quijano et al. [42] reported that depression interventions continue to be offered by participating agency offices. On the other hand, Butler and Quayle [63] noted the challenge of implementing assessment tools due to competing demands such as holidays, working part-time or being too busy, and Sin et al. [67] outlined one participant with difficulties in applying new knowledge owing to manpower shortages and constraints in time and space.

### Outcomes for older adults following the health needs assessment training

Seven studies [42, 43, 50, 53, 57, 66, 68] detailed outcomes for older adults following health needs assessment training for healthcare professionals. These outcomes included appropriate referrals, tailored interventions, fall prevention, symptom reduction, and improved overall function. Bruce et al. [50] highlighted that depressed older adults in the intervention group were more likely to receive appropriate referrals for mental health evaluation [50], aligning with findings where a minimal intervention significantly increased the detection of depression and further referrals to general practitioners [68]. The findings in two studies demonstrated that patients were referred to tailored resources designed to address their identified problems [53, 57]. Furthermore, Quijano et al. [42] revealed that older adults’ awareness of seeking help and the significance of physical activity for maintaining health improved. Quinlan and Ryer [43] stated that after providing care plans to 83 older adults, most implemented fall prevention strategies during a two-week follow-up call with 29 older adults, with only one fall reported. Piau et al. [66] identified the main causes of frailty and reported effective intervention recommendations and referrals [66]. Most physicians in Stolee et al. [55] reported better general function for older adults due to comprehensive geriatric assessment. Findings in two studies [42, 57] documented reductions in depression severity at the follow-up assessment due to appropriate referrals and interventions [42, 57], and significantly more older adults felt better and experienced pain reduction, followed by increased activity [42]. Additionally, Abbasi et al. [57] reported a slight improvement in health-related quality of life, including mobility, usual activities, pain/discomfort, and anxiety and depression, suggesting enhanced function [57].

## Discussion

This scoping review provides insights into training interventions for healthcare professionals assessing the physical, mental, and social health needs of older adults living at home. The analysis of 23 studies revealed that nearly all training interventions used a multifaceted training strategy combining teacher-driven and participant-engaging pedagogical approaches to teach healthcare professionals theoretical and practical knowledge. Health needs assessment training focuses on the skills needed to conduct single or multiple health needs assessments in older adults. Interprofessional collaboration was an essential part of most training interventions. Multiple studies noted that participants were satisfied with the training content and had increased confidence and competencies in health needs assessment and care planning. Studies have also reported a shift in work practices for health care professionals and some included results have shown improved health outcomes for older adults.

Our study revealed that most of the included studies blended the use of teacher-driven and participant-engaging pedagogical approaches [16, 42, 43, 50–65, 67, 68]. These approaches provide participants with confidence and competencies in health needs assessment [16, 41, 42, 51, 52, 54–60, 62–68]. Skilled healthcare professionals are crucial in facilitating the implementation of health assessments for older adults [69]. Lectures can be highly effective for learning, especially when they stimulate thinking and active engagement. Their effectiveness depends on the lecturer’s skill and can be improved by incorporating learner feedback, performance results, self-reflection, and peer feedback [70]. Another way to improve lecture quality is by including interactive elements such as practical skill training, following John Dewey’s “learning by doing” philosophy [32]. In our review, we identified fifteen studies that utilized participant-engaging approaches such as skills training, role-playing, simulations, hands-on training in real-world settings [16, 42, 43, 51, 52, 54, 55, 57–60, 62–65], and sixteen studies employed discussion, questioning and coaching [43, 51–56, 58–61, 63–65, 67, 68]. The integration of teacher-driven sessions, interactive training, and knowledge exchange resembles simulation training, which typically includes briefing, simulation exercises, and debriefing phases. These phases allow participants to reflect, enhance their learning, and deepen their educational experience [71]. Debriefing is a valuable tool for reflecting on and discussing experiences in training and real-world settings. This helps individuals and teams identify strengths, areas for improvement, and lessons learned, thereby enhancing learning and future performance [72]. However, effective debriefing relies on facilitators with strong skills to maximize learning outcomes [73].

The WHO advocates interprofessional simulation training to enhance healthcare professionals’ competencies and improve patient outcomes [2]. Even if several studies combined teacher-driven approaches, interactive training and knowledge exchange, our review included only one study utilizing simulation training [62]. Health needs assessment training for interprofessional teams was evident in eight of the studies included in our review [51, 55, 57–59, 61, 64, 65]. Such training has been proven to provide valuable insights into the health of older adults, leading to improved care delivery [74, 75], improved patient outcomes [76] and reduced hospitalization [74]. It can improve conflict management skills and team functioning [76] and play a critical role in equitably distributing community service resources [55]. Interprofessional simulation training is an engaging method for training clinical skills, procedures, teamwork, and communication in a safe, realistic environment [77]. It promotes critical thinking, reflection [78], and effective learning [79] enhancing the application of knowledge in clinical practice [80]. The use of participant engaging pedagogical approaches aligns with the sociocultural view of training, which emphasizes active engagement and collaboration in the learning process. It enables knowledge exchange and reflection, and participants can integrate their experiences with new information, internalize it, and construct new knowledge [32, 81]. Practical training such as simulations, can push participants out of their comfort zones, foster collaborative learning and enrich the educational experience [82]. However, to achieve optimal learning, it is crucial to balance skill development with an appropriate level of challenge as learners acquire new concepts. At the same time, temporary support from more experienced learners should be available. This balance is known as the zone of proximal development, which represents the space between a learner’s current skill level and their potential skill level with guidance. Tasks within this zone promote growth [83].

Our review reports a distinction in training content with a focus on assessing single versus multiple health needs in older adults. Ten studies [16, 41, 52–54, 58–60, 63, 68] focused solely on assessing depression. There is a strong correlation between late-life depression and reduced quality of life, as well as comorbidities such as physical illness, disability [58, 84] and physical frailty [85, 86]. However, single health need assessment training may inadvertently lead to the overlooking of broader health needs among older adults. A multiple health assessment of older adults is recommended [7], as it can serve as the foundation for developing holistic interventions to enhance overall health [10, 12, 87–89], promote health [90], foster positive health behaviors [91], and reduce frailty [92, 93]. Our review included eight studies [42, 51, 55–57, 61, 64, 66] providing training in physical, cognitive, mental, and social health needs assessment, alongside care planning on the basis of these assessments. Research indicates that both healthcare professionals and frail older adults participating in an interdisciplinary care approach were satisfied with the improved structure of care and appreciated the emphasis on health promotion [94]. On the other hand, a comprehensive health needs assessment is a multifaceted and complex intervention, with uncertainties surrounding its effectiveness and underlying mechanisms [95]. Some research findings indicate that there is no conclusive evidence that it reduces disability, prevents functional decline [96], impacts mortality, or supports independent living in older adults [97]. These results underscore the complexity and challenges in conducting and implementing comprehensive health needs assessments and tailoring interventions to promote health in older adults.

Our review revealed that almost all [16, 41–43, 50–61, 63–68] health needs assessment training programs included the use of assessment tools or checklists, leading to the identification of health needs. Only one of these studies reported that participants relied more on observation than on direct questioning when assessing depression [54]. Additionally, another study found that healthcare professionals using assessment tools felt that this approach led to asking overly personal and intrusive questions without first establishing trust or explaining the purpose of the assessment [98]. On the other hand, some older adults reported that using assessment tools made it difficult to discuss issues outside the predefined domains of the comprehensive health needs assessment [99]. Research indicates that current assessment practices heavily rely on professional judgment and intuition, and healthcare professionals in community settings often lack adequate knowledge and training regarding the health needs assessment of older adults [22, 100]. This can be seen as problematic because these professionals are ideally positioned to assess older adults early in their health trajectories [105]. Proper assessment in these settings can facilitate the early recognition of functional decline [101, 102] and vulnerability, enable timely intervention to mitigate frailty’s adverse effects [105], and support effective care planning [64]. Even if several healthcare professionals have endorsed the integration of frailty assessment tools into primary care [22], they need a simple, efficient assessment tool [105] that empowers them to identify older adults’ health needs [9, 88, 103, 104]. This is particularly critical due to the essential role that assessments play in equitably distributing community service resources [105]. As such, this review underscores the importance of educating healthcare professionals in community care to effectively assess the physical, mental, and social health needs of older adults. Furthermore, understanding the learning process of healthcare professionals [78], evaluating the effects of training [106], and establishing evidence-based standards for skills training are crucial for high-quality teaching [107]. Additionally, further research is necessary to assess the feasibility, effectiveness, and acceptability of interprofessional interventions targeting multiple health needs aimed at health promotion [90] and experiences using comprehensive health assessment tools [108].

### Methodological considerations

This review included studies employing various methods to obtain comprehensive insights into training healthcare professionals in assessing the health needs of older adults living at home [47]. We utilized a validated mixed-methods appraisal tool to assess the quality of the included studies [47, 49]. We did not include reporting on screening questions regarding the clarity of the research question or whether the collected data addressed the research questions, as our review focused exclusively on empirical studies. Additionally, we chose not to calculate an overall score from the ratings of each criterion, as this practice is discouraged. We provide an overview of each study’s quality by presenting the ratings of each criterion [49]. Our findings revealed that only one study met all the quality criteria, fifteen studies met four criteria, three studies met three criteria, and four studies met only one criterion. High-quality studies employ rigorous and robust methods, leading to reliable and valid findings [109]. While most studies met 3–4 quality criteria, they provide a relatively strong evidence base and offer valuable insights, although some concerns remain. Several studies did not meet the quality criteria for nonresponse bias or complete outcome data. It is crucial to describe and evaluate a low response rate for its potential impact, as this can limit the generalizability of findings [110]. Many studies also failed to account for confounders in their design and analysis. Confounding factors may bias results by distorting the interpretation of findings [49], masking actual associations or creating false associations, potentially leading to incorrect conclusions [111]. The randomization of study subjects and rigorous statistical analyses can mitigate the impact of confounding variables [112]. Nonetheless, conducting a quality assessment increases awareness of these biases and limitations, thereby enhancing our confidence in the study findings.

### Strengths and limitations

Our scoping review has several limitations. Initially, our search strategy involved the use of six databases and various relevant search terms related to training healthcare professionals in assessing the health needs of older adults. We excluded gray literature to focus on mapping existing published research and identifying any research gaps. The search was conducted by an experienced librarian. Despite our efforts to comprehensively map the research literature, we may have overlooked some studies. Second, our exclusion criteria, which encompassed, for example, general practitioners, students, and institutional settings, restricted the scope of the study. Additionally, we focused on health needs assessment, excluding studies that assessed the environment, an important factor in enabling older adults to stay at home as long as possible. However, based on the findings and limitations of the included studies, we believe our review provides valuable insights into the research context. These findings can inform future research, practice, policymaking, and the development of training programs for healthcare professionals in community settings to assess older adults’ health needs.

## Conclusion

Healthcare professionals require training in assessing physical, mental, and social health needs in older adults living at home to ensure tailored interventions that enhance their health and independence. Our study revealed that healthcare professionals were satisfied with the combination of participant-engaging and teacher-driven pedagogical approaches when training in physical, mental, and social health needs assessment. Such training is beneficial and strengthens healthcare professionals’ confidence and competency in assessment and care planning for older adults living at home. Additionally, some studies reported that following health needs assessment training, there was a shift in work practices and improved health outcomes for older adults. We suggest that health needs assessment training programs are valuable for improving health and care for older adults living at home and contribute to increased sustainability in healthcare.

Furthermore, we propose additional research on interprofessional simulation training for the structured assessment of multiple health needs in older adults, ensuring comprehensive coverage of all significant health issues in these assessments. We also recommend research on the implementation of such assessments and health promoting interventions.

## Electronic supplementary material

Below is the link to the electronic supplementary material.

Supplementary Material 1

Supplementary Material 2

Supplementary Material 3

## Data Availability

No datasets were generated or analysed during the current study.
