# Supplementary material for: Training healthcare professionals in assessment of health needs in older adults living at home: a scoping review
Source: BMC Med Educ. 2024 Sep 17;24:1019. doi: 10.1186/s12909-024-06014-9 (PMC11409792; doi:10.1186/s12909-024-06014-9)
Supplement: Supplementary file 1 — Supplementary Material 1 [file 12909_2024_6014_MOESM1_ESM.docx]

# Additional file 2

# Search history from Ovid Medline

Ovid MEDLINE(R) <1946 to September Week 5 2022>

1 geriatric assessment/ 31560

2 (geriatr* adj2 assess*).kf,ti,ab. 5521

3 ((assess* or checklist* or "check list*" or detect* or test* or measur* or screen* or identif* or recogniz* or scor* or mapping*) adj6 (functional adj2 (disabilit* or declin* or performance* or status or capacit* or impair* or limit* or problem*))).ti,ab. 18526

4 ((assess* or checklist* or "check list*" or detect* or test* or measur* or screen* or identif* or recogniz* or scor* or mapping*) and (functional adj2 (disabilit* or declin* or performance* or status or capacit* or impair* or limit* or problem*))).kf. 504

5 ((assess* or checklist* or "check list*" or detect* or test* or measur* or screen* or identif* or recogniz* or scor* or mapping*) adj6 ("at-risk" or risk factor* or cognitive function* or cognition or frailty or fragility or sensory or vision or hearing or sight or psychological health or (mental adj3 health) or physical status or physical wellbeing or physical well-being or loneliness* or depressi*)).ti,ab. 349460

6 ((assess* or checklist* or "check list*" or detect* or test* or measur* or screen* or identif* or recogniz* or scor* or mapping*) and ("at-risk" or risk factor* or cognitive function* or cognition or frailty or fragility or sensory or vision or hearing or sight or psychological health or (mental adj3 health) or physical status or physical wellbeing or physical well-being or loneliness* or depressi*)).kf. 15411

7 1 or 2 or 3 or 4 or 5 or 6 399622

8 Aged/ or "Aged, 80 and over"/ or Frail Elderly/ or Geriatrics/ or Geriatric Psychiatry/ or Geriatric Nursing/ or Geriatric Dentistry/ or "Dental Care for Aged"/ or "Health Services for the Aged"/ or (elder* or eldest or frail* or geriatri* or old age* or oldest old* or senior* or senium or very old* or septuagenarian* or octagenarian* or octogenarian* or nonagenarian* or centarian* or centenarian* or supercentenarian* or older people or older subject* or older patient* or older age* or older adult* or older man or older men or older male* or older wom#n or older female* or older population* or older person*).kf,ti,ab. 3583907

9 7 and 8 153829

10 (communit* or home* or residen* or (independent* adj2 living)).hw. 500607

11 (communit* or home* or residen* or (independent* adj2 living)).kf,ti,ab. 1269166

12 10 or 11 1465119

13 9 and 12 32164

14 ((education or training or develop* or competence* or learn* or teach* or capacity building or coaching or supervis* or simulati*) and (nurs* or careworker* or care worker* or physical therapist* or occupational therapist* or social worker*)).hw,kf.160189

15 ((education or training or develop* or competence* or learn* or teach* or capacity building or coaching or supervis* or simulati*) adj6 (nurs* or careworker* or care worker* or physical therapist* or occupational therapist* or social worker*)).ti,ab. 79440

16 ((education or training or develop* or competence* or learn* or teach* or capacity building or coaching or supervis* or simulati*) and (health* adj2 (professional or personnel or worker* or provider* or assistant*))).hw,kf. 59787

17 ((education or training or develop* or competence* or learn* or teach* or capacity building or coaching or supervis* or simulati*) adj6 (health* adj2 (professional* or personnel or worker* or provider* or assistant*))).ti,ab.25645

18 ((education or training or develop* or competence* or learn* or teach* or capacity building or coaching or supervis* or simulati*) and ((home* adj2 worker*) or (home* adj2 professional*))).hw,kf. 16

19 ((education or training or develop* or competence* or learn* or teach* or capacity building or coaching or supervis* or simulati*) adj6 ((home* adj2 worker*) or (home* adj2 professional*))).ti,ab. 128

20 14 or 15 or 16 or 17 or 18 or 19 252643

21 13 and 20 911

22 nursing home?.mp. [mp=title, book title, abstract, original title, name of substance word, subject heading word, floating sub-heading word, keyword heading word, organism supplementary concept word, protocol supplementary concept word, rare disease supplementary concept word, unique identifier, synonyms] 48551

23 21 not 22 510

24 21 and 22 401

25 ((education or training or develop* or competence* or learn* or teach* or capacity building or coaching or supervis* or simulati*) adj6 (nurs* or careworker* or care worker* or physical therapist* or occupational therapist* or social worker*)).ti. 32150

26 ((education or training or develop* or competence* or learn* or teach* or capacity building or coaching or supervis* or simulati*) adj6 (health* adj2 (professional* or personnel or worker* or provider* or assistant*))).ti. 3526

27 ((education or training or develop* or competence* or learn* or teach* or capacity building or coaching or supervis* or simulati*) adj6 ((home* adj2 worker*) or (home* adj2 professional*))).ti. 26

28 14 or 16 or 18 or 25 or 26 or 27 208917

29 7 and 8 and 12 and 28 558 Denne er lastet inn i EndNote

30 23 or 29 768

31 23 not 29 210
